# Effect of minute ventilation on central venous-to-arterial carbon dioxide difference

**DOI:** 10.1186/cc9464

**Published:** 2011-03-11

**Authors:** S Saengngammongkhol, A Wattanathum, A Wongsa

**Affiliations:** 1 Phramongkutklao Hospital, Bangkok, Thailand

## Introduction

The central venous-to-arterial carbon dioxide difference (P(cv-a)CO_2_, dPCO_2_) is a global index of tissue perfusion. A normal dPCO_2 _indicates cardiac output (CO) is high enough to wash out CO_2 _production from peripheral tissues. An increased dPCO_2 _suggests that CO is not high enough with respect to global metabolic conditions. PCO_2 _depends on alveolar ventilation. We hypothesized that minute ventilation (MV) has an effect on dPCO_2_.

## Methods

A prospective experimental, pilot study was performed on 19 patients admitted to a medical ICU with septic shock between August 2010 and November 2010. All patients were intubated and on a mechanical ventilator with continuously monitoring end-tidal CO_2_, central venous pressure (CVP), blood pressure (BP), and CO. Mechanical ventilator was set consecutively in three steps every 30 minutes (T0, T30, T60) by increasing the respiratory rate (RR) for MV of 8 l, 15 l, and 8 l, respectively. Tidal volume, RR, MV, auto-PEEP, CO and dPCO_2 _were recorded at each step of MV changed for all patients.

## Results

Patients' age and APACHE II scores were 67.3 ± 13.2 years and 24.4 ± 6.6, respectively. There was a significant difference between the dPCO_2 _between T0 and T30 (3.5 ± 3.5 vs. 5.9 ± 2.0, *P *= 0.04) (Table 1). Moreover, there was significantly decreased CO from T0 to T30 (5.1 ± 1.4 vs. 4.5 ± 1.11, *P *= 0.002) and, also, T30 and T60 (4.5 ± 1.1 vs. 5.0 ± 1.3, *P *= 0.009). Auto-PEEP values were inversely correlated with decreased CO (*P *< 0.001) at T30.

**Table 1:** 

				*P *value		
				
Variable	T0	T30	T60	T0 vs. T30	T30vs. T60	T0 vs. T60
dPCO_2_	3.5 ± 3.5	5.9 ± 2.0	4.8 ± 2.1	0.040	0.15	0.49
CO	5.1 ± 1.4	4.5 ± 1.1	5 ± 1.3	0.002	0.009	0.97

## Conclusions

Minute ventilation had an effect on dPCO_2 _by reduced CO due to development of auto-PEEP. The dPCO_2 _should be measured during normal minute ventilation without auto-PEEP.
